# EVALUATING IN-HOME ASSISTIVE TECHNOLOGY FOR DEMENTIA CAREGIVERS: CHALLENGES AND OPPORTUNITIES

**DOI:** 10.1093/geroni/igae098.1415

**Published:** 2024-12-31

**Authors:** Kuan-Hua Chen, Julian Scheffer, Claire Yee, Jennifer Merrilees, David Moss, Brian Hinderberger, Gene Wang, Robert Levenson

**Affiliations:** University of Nebraska Medical Center, Elkhorn, Nebraska, United States; University of California Berkeley, Berkeley, California, United States; Mayo Clinic, Scottsdale, Minnesota, United States; University of California San Francisco, San Francisco, California, United States; Care Daily Company, Kirkland, Washington, United States; Care Daily Company, Kirkland, Washington, United States; Care Daily Company, Kirkland, Washington, United States; University of California Berkeley, Berkeley, California, United States

## Abstract

Dementia caregivers are at heightened risk for developing mental and physical health problems. Interventions designed to protect caregiver health often create additional demands on time and energy (e.g., exercise programs). Technology-based interventions (e.g., remote monitoring) can be effective, scalable, and do not add greatly to caregiver burden. Through a collaboration with a local technology company, Care Daily, we conducted two randomized controlled trials to evaluate People Power Caregiver (PPCg), a system of sensors in the home connected to cloud-based AI that alerts caregivers about worrisome deviations from normal patterns (e.g., care-recipient’s falls, wandering). Across two studies (study 1: 9-month duration with professional installation of PPCg; study 2: 6-month duration with caregiver self-installing the PPCg using remote tech support), 284 caregivers were recruited; 261 completed initial study deployment (e.g., system installation); and 235 completed the entire study protocol. Use of PPCg was associated with (a) reduced caregiver anxiety in study 1; (b) greater protection from declines in sleep efficiency in studies 1 and 2. These findings underscore the promise of technology-based intervention to mitigate health problems in dementia caregivers. However, challenges and complexities in recruitment (e.g., remote consenting both caregivers and care recipients), retention (e.g., due to changes in home environment or caregiving status), system installation (e.g., working with caregivers with less experience using technology), data quality assurance (e.g., invalid sensor data) as well as our efforts to overcome these challenges have been instructive. Lessons learned from these studies can provide useful guidance for future large-scale, technology-based interventions for dementia caregivers.

